# A Randomized, Double-Blind, Active Control, Multicenter, Phase 3 Study to Evaluate the Efficacy and Safety of Liztox^®^ versus Botox^®^ in Post-Stroke Upper Limb Spasticity

**DOI:** 10.3390/toxins15120697

**Published:** 2023-12-12

**Authors:** Dong Hyun Ye, Min Ho Chun, Yoon Ghil Park, Nam-Jong Paik, Shi-Uk Lee, Seung Don Yoo, Deog Young Kim

**Affiliations:** 1 Department of Rehabilitation Medicine, Asan Medical Center, University of Ulsan College of Medicine, Seoul 05505, Republic of Korea; ydh7342@amc.seoul.kr; 2 Department of Rehabilitation Medicine, Gangnam Severance Hospital, Yonsei University College of Medicine, Seoul 06273, Republic of Korea; drtlc@yuhs.ac; 3 Department of Rehabilitation Medicine, Seoul National University College of Medicine, Seoul National University Bundang Hospital, Seongnam 13620, Republic of Korea; njpaik@snu.ac.kr; 4 Department of Rehabilitation Medicine, SMG-SNU Boramae Medical Center, Seoul National University College of Medicine, Seoul 07061, Republic of Korea; paindoc@snu.ac.kr; 5 Department of Rehabilitation Medicine, Kyung Hee University Hospital at Gangdong, Kyung Hee University College of Medicine, Seoul 05278, Republic of Korea; kidlife@khu.ac.kr; 6 Department of Medicine (AgeTech-Service Convergence Major), Kyung Hee University, Seoul 05278, Republic of Korea; 7 Department and Research Institute of Rehabilitation Medicine, Yonsei University College of Medicine, Seoul 03722, Republic of Korea; kimdy@yuhs.ac

**Keywords:** spasticity, botulinum toxin, upper extremity, stroke

## Abstract

Botulinum toxin type A (BTX-A) injection is a commonly used therapeutic intervention for upper limb spasticity in stroke patients. This study was designed as a randomized, active-drug-controlled, double-blind, multicenter, phase 3 clinical trial to evaluate the safety and efficacy of Liztox^®^ in comparison to onabotulinum toxin A (Botox^®^) for individuals with post-stroke upper limb spasticity. The primary outcome was the alteration in wrist flexor muscle tone from the initial assessment to the fourth week, evaluated using the modified Ashworth scale (MAS). Secondary outcomes included MAS score changes for the wrist at weeks 8 and 12 from baseline; MAS score changes for finger and elbow flexors; and changes in the Disability Assessment Scale (DAS), Subject’s Global Assessment (SGA), the Investigator’s Global Assessment (IGA), and Caregiver Burden Scale (CBS) at weeks 4, 8, and 12 from baseline. The MAS score for wrist flexor spasticity decreased by −1.14 ± 0.59 in the Liztox^®^ group and −1.22 ± 0.59 in the Botox^®^ group from baseline to week 4. The difference [97.5% confidence interval (CI)] between the test and control groups was 0.08 [−∞, 0.26], confirming the non-inferiority of the test group compared to the control group. Furthermore, there were consistent improvements in the IGA, SGA, and CBS scores across all assessment intervals, with no statistically significant variances detected between the two groups. No safety-related concerns were reported during the study. In conclusion, Liztox^®^ injection proved to be a secure and efficacious intervention for managing upper extremity spasticity in post-stroke patients.

## 1. Introduction

Stroke is the leading cause of death and disability worldwide, presenting a significant burden on individuals and society as a whole [1,2,3]. Among stroke patients, approximately 20–40% experience the debilitating effects of spasticity, which greatly diminishes their quality of life and places added strain on their caregivers [4,5,6]. Spasticity affecting the hands and wrists can be particularly troublesome as it can disrupt tasks such as dressing, personal hygiene, and other everyday activities. Thus, it becomes crucial to alleviate the challenges posed by spasticity following a stroke. Optimized spasticity management can reduce the level of disability and increase the quality of life of stroke patients.

Spasticity management involves a range of treatments including oral medication, intrathecal baclofen treatment, and physical therapy [7]. However, these approaches have limitations. Oral medication, for instance, often leads to side effects, prompting 12–38% of patients to discontinue its use [7]. Furthermore, invasive treatments such as intrathecal baclofen and phenol are effective in controlling muscle tone and last longer than botulinum toxin, but they can cause pain [8,9]. In cases of localized spasticity, botulinum toxin offers a valuable alternative by blocking acetylcholine release at the neuromuscular synapse, providing relatively prompt relief (within 3–7 days) [9,10,11]. This treatment is frequently used for stroke patients who struggle with oral muscle relaxants, as these medications can cause drowsiness, temporary cognitive impairment, and decreased arousal levels [7,12].

Liztox^®^ (Huons Biopharma, Seongnam, Korea) is a newly developed Botulinum toxin type A (BTX-A). The in vivo test measures the response to electrical stimulation after the administration of Liztox^®^ to the hind limb muscles of mice, resulting in a dose-dependent decrease in the electrical stimulation response similar to that observed with onabotulinum toxin A [13,14]. In a phase 1 clinical trial to prove the safety and efficacy of Liztox^®^ in patients with muscle spasticity in the upper extremities after stroke, compared with the safety profile of the existing BTX-A treatment, no specific side effects were identified due to the administration of Liztox^®^ [13]. Notably, significant improvements in spasticity, degree of disability, and caregiver burden were observed. The observed effect endured for 12 weeks after Liztox^®^ injection, consistent with the previous studies [10,15,16,17]. These findings suggest that Liztox^®^ is a safe treatment option for improving muscle tone in individuals with spasticity of the upper extremity muscles after a stroke.

The objective of this study was to investigate the efficacy and safety of the administration of Liztox^®^ and Botox^®^ in adult patients requiring treatment for upper extremity muscle spasticity after a stroke. The comparative evaluation aimed to demonstrate the non-inferiority of Liztox^®^ compared to Botox^®^.

## 2. Results

**Figure 1:**
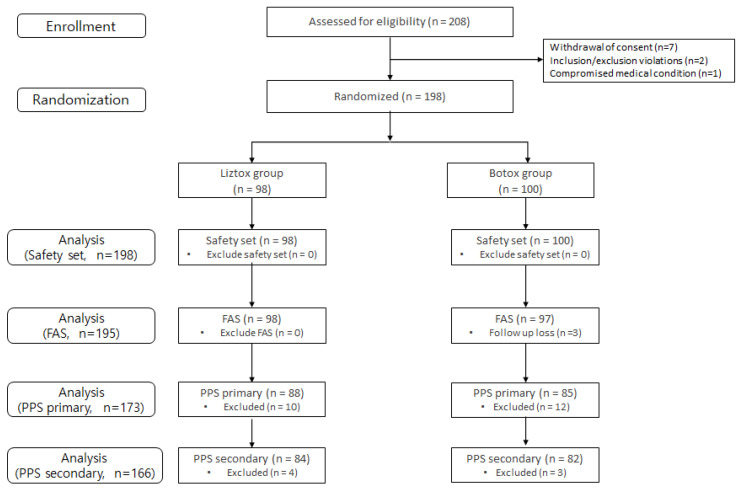
Flowchart of the study. FAS, full analysis set; PPS, per protocol set.

A total of 195 subjects (treatment: 98, control: 97) who received the studied medication and had confirmed efficacy endpoints were included in the FAS. Of the patients included in the FAS, 173 (88 in the treatment arm and 85 in the control arm) who completed at least 4 weeks of study without a major protocol violation were included in the PPS-Primary, and 166 (84 in the treatment arm and 82 in the control arm) who completed the study without a major protocol violation were included in the PPS-Secondary. Figure 1 displays the study’s flowchart and provides demographic information along with the baseline characteristics of the participants. The age of the patients included in the FAS was 59.31 ± 11.89 years, with a higher proportion of males (68.72% male, 31.28% female), and the BMI was 24.77 ± 3.25 kg/m^2^. The duration of post-stroke upper extremity muscle spasticity was 111.56 ± 78.8 months, and all patients included in the FAS had not received BTX-A within 3 months prior to screening. The most common DAS assessment goal item set at the screening visit was “Limb position” for 140 (71.79%) patients, followed by “Hand hygiene” for 38 (19.49%), “Dressing” for 9 (4.62%), and “Pain” for 8 (4.10%), with no subsequent changes in goal items. There were no statistically significant differences observed in patient demographics and baseline characteristics between the treatment groups (Table 1).

### 2.1. Primary Outcome

Table 2 displays the modifications in MAS scores for the wrist flexor muscle 4 weeks following the intervention. In the PPS, the mean (SD) change in the MAS scores for wrist flexor muscle tone from baseline to week 4 was −1.14 (0.59) points in the treatment group and −1.22 (0.59) points in the control group, with a difference [97.5% CI] of 0.08 [−∞, 0.26]. The upper limit of the confidence interval (0.26) was less than the margin of non-inferiority, confirming the non-inferiority of the treatment group compared to the control group.

### 2.2. Secondary Outcome

#### 2.2.1. Change in MAS Scores for Wrist Flexor Muscle at 8 and 12 Weeks and Response Rates

**Figure 2:**
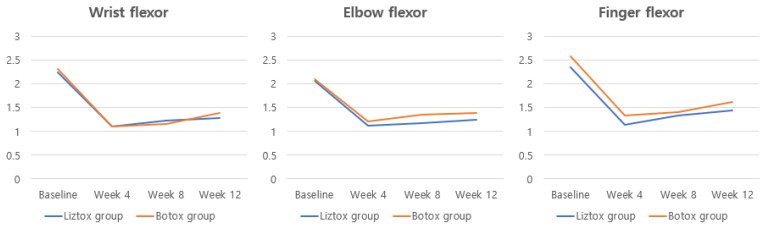
Changes in the modified Ashworth scale for each muscle group after BTX-A injections.

In the PPS analysis, the mean (SD) change in the MAS scores for the wrist flexor muscle from baseline to 8 and 12 weeks was −1.02 (0.56) and −0.96 (0.62) points, respectively, in the Liztox^®^ group and −1.18 (0.60) and −0.94 (0.64) points, respectively, in the Botox^®^ group (Table 3, Figure 2). Response rates for wrist flexors in the Liztox^®^ group were 82.1%, 76.8%, and 70.4% at 4, 8, and 12 weeks, respectively, and 85.4%, 81.0%, and 67.5% in the Botox^®^ group (Table 4).

#### 2.2.2. Change in MAS Scores for Elbow Flexor and Finger Flexor Muscle Tone at 4, 8, and 12 Weeks

In the PPS, there were no statistically significant differences observed in the mean (SD) change from baseline in MAS scores for elbow flexor and finger flexor muscle tone between the treatment groups at 4, 8, and 12 weeks following the intervention (Table 3, Figure 2). Response rates, defined as a decrease in the MAS score at the treatment site of at least one point from baseline, ranged from approximately 50 to 80%. There were also no statistically significant differences between the treatment arms at any time point (Table 4).

#### 2.2.3. Change in Scores of the Target Endpoints of the DAS at 4, 8, and 12 Weeks

In the PPS analysis, the mean (SD) changes in scores of the targeted endpoints of the DAS from baseline to 4, 8, and 12 weeks after study drug administration are presented in Table 5. Among the assessment items, statistically significant improvements in the DAS ‘Total’ and ‘Hand Hygiene’ items from baseline to week 12 (*p*-value = 0.0047, 0.0048) were found in the treatment group compared to the control group.

#### 2.2.4. Global Assessment (IGA, SGA) and CBS at 4, 8, and 12 Weeks

In the PPS, the means (SD) of investigator- and patient-rated overall improvement (IGA, SGA) at 4, 8, and 12 weeks after study drug administration did not show statistically significant differences between treatment groups (Table 6). In the IGA, the percentages of subjects scoring five or higher at each time point in the Liztox^®^ group were 90.5%, 84.2%, and 85.2% at 4, 8, and 12 weeks, respectively, and 86.6%, 83.5%, and 78.8% in the Botox^®^ group (*p* > 0.05). In the SGA, the percentages of subjects scoring five or higher in the Liztox^®^ group were 69.1%, 69.5%, and 61.7% at 4, 8, and 12 weeks, respectively, and 64.6%, 58.2%, and 57.5% in the Botox^®^ group (*p* > 0.05). The Liztox^®^ group showed a higher percentage of responders by physician and caregivers, but it was not statistically significant (*p* > 0.05). The mean (SD) change in the CBS also did not show a statistically significant difference between the treatment groups at any time point (Table 7). 

### 2.3. Safety Assessment

Of the 198 patients in the safety set, 24 (12.12%) experienced 46 treatment-emergent adverse events (TEAEs) and 3 (1.52%) experienced 3 adverse drug reactions (ADRs), with no statistically significant differences in the incidence of TEAEs and ADRs between the treatment arms (Table 8 and Table 9). The most common TEAEs were ‘COVID-19’, with seven cases in seven patients (3.54%); followed by ‘Alanine aminotransferase increased’ and ‘Aspartate aminotransferase increased’, with four cases each in two patients (1.01%); followed by ‘Pyrexia’ and ‘Hand fracture’ with two cases each; and ADRs included ‘Asthenia’, ‘Headache’, and ‘Pruritus’, with one case each (0.51%). Serious adverse events (SAEs) occurred in four subjects (2.02%) to five cases (‘Cerebral infarction’, ‘Cerebrovascular accident’, ‘Transient ischemic attack’, ‘Febrile neutropenia’, ‘Vomiting’), none of which were related to the study medication, with three of the five reported as ‘recovered/resolved’ and two as ‘recovering/resolving (Table 9). There were no statistically significant differences in the incidence of serious adverse events between the treatment arms. There were no acute adverse events within 30 min of study drug administration, adverse events that resulted in the discontinuation of study drug administration, or deaths during the study. Clinical laboratory tests showed three clinically significant abnormal changes in one subject, all of which were reported as adverse events (“alanine aminotransferase increased” and “aspartate aminotransferase increased”). All three AEs were not causally related to the study drug and did not represent serious adverse events. No clinically significant abnormal changes were observed after the study drug administration. Physical examinations revealed clinically significant changes in two subjects that were not reported to be related to the study drug, as the changes were attributed to a wrist fracture and stroke, respectively. The adverse events were followed up with pharmacological therapy, the reported adverse events were considered “recovering/resolved”, and follow-up was discontinued. An assay to detect the formation of neutralizing antibodies was positive in one participant in the safety set (study arm) at 12 weeks after the administered dose. No other laboratory abnormalities were reported and no female participants experienced pregnancy during the study.

## 3. Discussion

This phase 3 clinical trial is the first randomized, double-blind, active-drug-controlled study to compare the efficacy and safety of Liztox^®^ with Botox^®^ in patients with upper limb spasticity after a stroke. The results showed the non-inferior efficacy and safety of a newly developed BTX-A (Liztox^®^) compared with Botox^®^ for the treatment of post-stroke upper limb spasticity.

BTX-A typically reaches its maximum effectiveness within about a week and tends to diminish in efficacy after 3 to 4 months [17,18]. Week 4 was chosen as the primary endpoint assessment time because this is when the BTX-A effect reaches its maximum optimization following the treatment. The MAS scores at the wrist flexor decreased by −1.14 ± 0.59 and −1.22 ± 0.59 from baseline to week 4 in the Liztox^®^ and Botox^®^ groups, respectively (Table 2). These changes are comparable to previous results, which ranged from −1.1 to −1.5. The changes in the MAS scores in the elbow flexor at 4 weeks (Liztox^®^: −0.98, Botox^®^: −0.90) and the finger flexor (Liztox^®^: −1.23, Botox: −1.26) were also similar to those in previous studies (Table 3) [10,19].

Spasticity is characterized by an increase in muscle tone that depends on speed or a tonic stretch reflex. This condition can hinder movement and is often associated with discomfort or pain. However, using only the MAS may not adequately reflect the extent of functional deficits and disability caused by spasticity, necessitating a comprehensive clinical evaluation. Therefore, as secondary outcomes, we evaluated disability from spasticity, upper limb function using the DAS and CBS, and overall treatment effectiveness using the SGA and IGA. In the current study, the DAS scores displayed notable improvement throughout the entire 12-week duration, aligning with prior research findings indicating that upper limb function, as assessed using the DAS, improved following the administration of BTX-A (Table 5) [16,20,21]. The overall evaluation conducted by the investigator and the patients consistently exceeded an average rating of five points across all time intervals (Table 6). This outcome suggests that the majority of patients demonstrated improvement in activities of daily living involving the upper limb, such as dressing and hand hygiene, as well as a reduction in pain. Additionally, they expressed satisfaction with the treatment.

Although there was a general decline in treatment effectiveness over time, the influence of BTX-A on the MAS scores remained consistent for 12 weeks after the intervention. When comparing the Botox^®^ group with the Liztox^®^ group in terms of DAS, IGA, and SGA scores and response rates at 4, 8, and 12 weeks, no statistically significant differences in treatment efficacy were observed. In terms of the maintenance of effect throughout the 12-week follow-up period, no differences were observed between the two groups, and these findings are consistent with the results of previous studies [16,20,21,22]. Notably, there was a statistically significant improvement in the DAS ‘Total’ and ‘Hand Hygiene’ category scores at 12 weeks in the Liztox^®^ group compared to the control group (*p*-value = 0.0047, 0.0048) (Table 5). The improvement in upper limb function after BTX-A injection is controversial. However, the significant improvement in DAS scores at 12 weeks in our study compared to the control group is encouraging. Numerous studies have documented an overall enhancement in upper limb function following botulinum toxin treatment, particularly with regard to passive or basic functions. Although spasticity is thought to be a factor in diminished active arm function, the exact connection between spasticity and motor performance remains a topic of debate [17,23,24,25]. While some argue that spasticity plays a significant role in diminished upper limb function, others contend that the primary issue lies in motor weakness. Physicians should contemplate the functional aspects and weigh the benefits and costs when deciding which muscles to administer BTX-A to. Future research will be needed to explore active upper limb function following BTX-A treatment and the long-term benefits of BTX-A.

A notable improvement in MAS grade following BTX-A injection has been consistently reported in various studies, and our study showed similar efficacy with Liztox^®^. Previous studies have shown a dose-dependent, effective reduction in spastic muscle tone, improvement in passive range of motion (PROM) and passive function (as measured by the DAS), and a reduction in caregiver burden related to handling the affected limb after BTX-A injection [17,25,26,27,28,29]. However, there has been ongoing debate about how changes in MAS scores translate into tangible, real-world improvements in patients’ lives [30,31]. While most studies investigating the efficacy and safety of BTX-A have emphasized a reduction in muscle tone, some studies have prioritized functional improvement as the primary measure [27,30,31,32]. Cardoso et al. showed that the careful selection of muscles and individualized doses of BTX-A could improve the functionality of certain patients after a stroke [30,33]. McCrory et al. reported that BTX-A was safe and effective in reducing upper limb spasticity, but did not result in a change in quality of life using standardized measures [34]. However, the treatment did seem to help achieve patient-centered goals. The effectiveness of BTX-A in improving active upper limb function, such as reaching and grasping, is still uncertain. Nevertheless, it is clinically important for patients with chronic post-stroke conditions, as it provides benefits in patient-oriented outcomes such as basic upper limb function, pain, and satisfaction [17]. Future research is needed to determine whether Liztox^®^ injection can improve functional scales in post-stroke spasticity patients.

Regarding safety, all adverse events documented during this study were categorized as mild or moderate, and no adverse events resulting in fatalities were recorded. Furthermore, there were no notable disparities in the safety assessment between the Liztox^®^ and Botox^®^ groups. In the antibody formation test, only one patient in the Liztox^®^ group developed antibodies to BTX-A at 12 weeks post injection. Our study did not find a difference in treatment response due to neutralizing antibodies, but it is imperative to closely monitor the formation of neutralizing antibodies as their development may lead to treatment failure [35]. Although our study included patients who had not previously received BTX-A, certain patients may require subsequent injections in the clinical management of post-stroke spasticity. Gracies et al. have documented that repeated rounds of abobotulinumtoxinA injections show consistent improvement in active upper limb function [36,37]. There is a growing trend towards the use of multiple injections for maximum therapeutic effect, although the efficacy and safety profile of this treatment is still controversial [36,37,38]. Further research is needed to determine long-term safety concerns and the time period over which therapeutic effects wear off. This will provide valuable information on the maximum therapeutic effect of repeated injections.

### Limitations

This study had some limitations. First, we did not include a placebo group for comparison. It is important to note that previous placebo-controlled trials have already established the efficacy of BXT-A injections, which have become a standard treatment for post-stroke upper limb spasticity. Consequently, it was deemed ethically inappropriate to administer placebo injections to these patients. Second, the study duration was relatively short. It is necessary to conduct long-term follow-up assessments to evaluate the sustained effects of the treatment. Finally, patients were included in this trial if they were receiving muscle relaxants or anti-spasticity drugs at a constant dose, and if they had been receiving physiotherapy for more than 4 weeks at the time of screening and were not expected to change during the trial. The stable dose of medication may not have been strictly monitored, and the short-term effect of physiotherapy at the time of assessment may be a potential bias.

## 4. Conclusions

In conclusion, the study demonstrated the non-inferiority of Liztox^®^ compared to Botox^®^ for improving wrist flexor muscle tone in patients with upper extremity spasticity after stroke, with both treatments showing similar efficacy at reducing muscle tone and improving disability at the site of upper extremity spasticity through to week 12, and no specific safety profile was identified with Liztox^®^ administration. There was also a statistically significant improvement in the DAS ‘Total’ and ‘Hand Hygiene’ category scores after 12 weeks in the Liztox^®^ group compared to the control group. This study confirms that Liztox^®^ injection is a safe and effective agent for the treatment of upper extremity spasticity in patients with post-stroke upper extremity muscle spasticity.

## 5. Material and Methods

### 5.1. Study Design

This study was designed as a randomized, double-blind, active-drug-controlled, multicenter, phase 3 trial conducted in six university hospitals (Asan Medical Center, Gangnam Severance Hospital, Seoul National University Bundang Hospital, SMG-SNU Boramae Medical Center, Kyung Hee University Hospital at Gangdong, Sinchon Severance Hospital) in Seoul, Korea between September 2021 and March 2023. This research adhered to the study protocol, followed the Good Clinical Practice guidelines, and complied with all relevant regulatory prerequisites. It received approval from the Ministry of Food and Drug Safety and the Institutional Review Boards of the participating institutions. Additionally, written informed consent was obtained from all participants prior to their enrollment. After the subject had voluntarily consented in writing to participate in this clinical trial, the investigator conducted a screening of the subject. After that, only test subjects who met the selection/exclusion criteria were enrolled.

### 5.2. Randomization

In this clinical trial, an independent statistician not directly involved in the study used SAS^®^ 9.4 to generate a randomization table. The eligible patients were then assigned random allocation numbers at a 1:1 ratio to the Liztox^®^ or Botox^®^ treatment group using a stratified block randomization method. An Interactive Web Response System was utilized to assign these random allocation numbers to the enrolled subjects in the order of their registration, along with their screening numbers, serving as identifiers throughout the duration of the clinical trial. An independent pharmacist handled the dilution and drug addition into empty vials. Filled vials and syringes were delivered to the investigator in charge of administration and blinded. To maintain double blindness, we used the assignment code during randomization so that test subjects were distinguished only by the assignment code, and the assignment for each group remained confidential until the end of the clinical trial.

### 5.3. Participants

The study recruited individuals who had significant spasticity in their upper limbs following a stroke. Inclusion criteria for the study included being 19 years of age or older, having experienced a stroke at least 24 weeks prior, demonstrating a minimum of 2 points of spasticity in the wrist flexors as measured on the MAS, showing at least 1 point of spasticity in one or more elbow flexors or finger flexors on the MAS, and reporting a rating of 2 or higher on the Disability Assessment Scale (DAS) for a specific functional disability item such as hygiene, dressing, limb position, or pain [39,40]. The exclusion criteria were a history of hypersensitivity to ingredients included in clinical trial drugs, phenol or alcohol injection or surgery in the target limb within 24 weeks, concurrent treatment with intrathecal baclofen, neuromuscular junction disorder, and skin abnormalities such as infection, skin disease, or scar at the site to be administered. Physical, occupational, and splinting therapy, as well as muscle relaxants and benzodiazepine medications, needed to remain consistent in dosage and regimen from one month before the screening process and throughout the study.

### 5.4. Intervention

Eligible participants were randomly assigned to the experimental group (Liztox^®^) or the control group (Botox^®^). They were then scheduled for visits every 4 weeks to assess the effectiveness and safety of the interventions over 12 weeks. Experienced physicians utilized ultrasound or electromyography examinations to determine the location of the muscles. They administered the injections using an appropriate needle length based on the depth of the muscle tissue at the administration site, following the recommended dosages listed in the Supplementary Figure S1. Within the recommended dosages, the physician determined the target muscles and specific injection doses based on the level of spasticity. The wrist flexors, including the flexor carpi radialis (15–60 U, 1–2 sites) and the flexor carpi ulnaris (10–50 U, 1–2 sites), were injected if the MAS score was 2 or more. Furthermore, elbow flexors and finger flexors, including the flexor digitorum sublimis (15–50 U, 1–2 sites), the flexor digitorum profundus (15–50 U, 1–2 sites), and the biceps brachii (100–200 U, up to 4 sites), were injected if the MAS score was 1 or more. However, the total dose of BTX-A did not exceed 360 units.

### 5.5. Assessment

Following an evaluation that encompassed gathering demographic information (age and sex), reviewing medical records, assessing prior medications or therapies, examining previous BTX-A injections, measuring vital signs, conducting physical examinations, and conducting laboratory tests on blood and urine, including a pregnancy test, suitable individuals were recruited. During the treatment session, participants received BTX-A injections into the specific muscles within 2 weeks of the initial screening. Follow-up evaluations took place at 4, 8, and 12 weeks following the BTX-A injection.

### 5.6. Efficacy Measures

The primary outcome measurement was the change from the baseline MAS score at the wrist flexors at 4 weeks after injection [40]. The secondary outcome assessment involved evaluating the change from the baseline MAS score at the wrist flexors at 8 and 12 weeks; at the elbow flexor and the finger flexor at 4, 8, and 12 weeks; an alteration in DAS score relative to baseline at 4, 8, and 12 weeks; a shift in the Caregiver Burden Scale (CBS) ratings by the caregiver from baseline at 4, 8, and 12 weeks; a response rate of elbow flexor and finger flexor at 4, 8, and 12 weeks; Investigator’s Global Assessment (IGA) at 4, 8, and 12 weeks; and Subject’s Global Assessment (SGA) at 4, 8, and 12 weeks [29,39,40].

### 5.7. Safety Measures

Throughout the study period, all participants were monitored for any unexpected events or symptoms. Physicians assessed adverse events for their possible connection to the medication, along with evaluating their severity. During each visit, physical examinations and vital signs were conducted, with baseline, 4-, and 12-week laboratory tests. An antibody test (Mouse Lethality assay) was conducted at baseline and 12 weeks [41,42]. Adverse events, serious adverse events, the number of subjects by treatment group, incidence rate, and the number of occurrences after drug administration were monitored.

### 5.8. Statistical Analysis

The efficacy evaluation analysis was primarily conducted on the per protocol set (PPS), with supplementary analysis performed on the full analysis set (FAS). The FAS included subjects who were enrolled in the clinical trial, received the investigational medicinal product at least once, and for whom information on the primary efficacy evaluation results after baseline could be obtained. The FAS was analyzed according to the randomly assigned treatment group. The PPS was defined as the subset of subjects included in the FAS who completed the clinical trial without any major protocol deviations. Statistical analysis was using SAS^®^ version 9.4 (SAS Institute, Cary, NC, USA).

For the primary outcome analysis, the upper limit of the 97.5% one-sided confidence interval for the difference value (test group–control group) of the MAS score change for wrist flexor muscle tone at 4 weeks after drug administration compared to the baseline is presented. If the difference was smaller than the margin of non-inferiority, it was determined that the test group had demonstrated non-inferiority compared to the control group. In addition, the difference between the two groups was compared using the Wilcoxon rank sum test or the two-sample t-test for the MAS score change of the wrist flexor for each administration group.

For the secondary outcome measurements to evaluate the change in DAS and CBS scores from baseline, descriptive statistics for each administration group (number of subjects, median, mean, standard deviation, minimum, maximum) were presented and the difference between the two groups was compared using the two-sample *t*-test or Wilcoxon rank sum test. In addition, the Chi-square test or Fisher’s exact test was used to evaluate the response rate of the elbow and finger flexor. Descriptive statistics for each treatment group are presented for the overall improvement at 4, 8, and 12 weeks. The response was defined as a decrease of 1 point or more on the MAS of the administration site from the baseline. Also, Fisher’s exact test or Chi-square test was used to assess the Subject’s Global Assessment at 4, 8, and 12 weeks after drug administration. The descriptive statistics are presented for each treatment group and the difference between the two groups is presented as the change in scores for each of the four CBS factors (washing hands, clipping nails, wearing clothes, washing under the armpits) evaluated by the caregiver at 4, 8, and 12 weeks after the intervention compared to the baseline.

## Figures and Tables

**Table 1:** Demographics and baseline characteristics of patients (full analysis set).

	Liztox Group (*n* = 98)	Botox Group (*n* = 97)	*p*-Value
Age (years)	59.2 ± 12.6	59.4 ± 11.2	0.8420
Sex, *n* (%)			0.9155
Male	67 (68.4)	67 (69.1)	
Female	31 (31.6)	30 (30.9)	
Height (cm)	164.8 ± 7.7	165.9 ± 8.2	0.3566
Body weight (kg)	67.9 ± 11.0	68.0 ± 12.1	0.8441
BMI (kg/m^2^)	24.9 ± 3.4	24.6 ± 3.1	0.6782
Duration of spasticity (months)	116.7 ± 80.3	106.4 ± 77.4	0.4196
Previous BTX-A administration *, *n* (%)			-
Yes	0 (0)	0 (0)	
No	98 (100)	97 (100)	
DAS items, *n* (%)			0.6039
Hand hygiene	21 (21.4)	17 (17.5)	
Dressing	3 (3.1)	6 (6.2)	
Limb position	71 (72.5)	69 (71.1)	
Pain	3 (3.1)	5 (5.2)	

* Botulinum toxin administration within 3 months prior to screening.

**Table 2:** Change from baseline in MAS scores for wrist flexor at 4 weeks (PPS).

	Liztox^®^ Group (*n* = 88)	Botox^®^ Group (*n* = 85)	Total (*n* = 173)
Baseline			
*n*	88	85	173
Mean (SD)	2.25 (0.44)	2.32 (0.47)	2.28 (0.45)
Median	2.00	2.00	2.00
Min, Max	2.00, 3.00	2.00, 3.00	2.00, 3.00
Week 4			
*n*	88	85	173
Mean (SD)	1.11 (0.56)	1.10 (0.52)	1.11 (0.54)
Median	1.00	1.00	1.00
Min, Max	0.00, 3.00	0.00, 2.00	0.00, 3.00
Change from baseline at week 4 [1]			
*n*	88	85	173
Mean (SD)	−1.14 (0.59)	−1.22 (0.59)	−1.18 (0.59)
Median	−1.00	−1.00	−1.00
Min, Max	−3.00, 0.00	−3.00, 0.00	−3.00, 0.00
Difference [97.5% confidence interval] *	0.08 [−∞, 0.26]		
*p*-value [2]	0.3013 [b]		

SD = standard deviation, Min = minimum, Max = maximum. * If the upper boundary of the confidence interval was less than or equal to 0.45, the test group was proven non-inferior to the control group. [1] Change = week 4—baseline. [2] *p*-value for comparisons between treatment groups. * The level of significance was set at *p* < 0.05.

**Table 3:** Change in the MAS scores for wrist, elbow, and finger flexor muscle tone at 4, 8, and 12 weeks (PPS).

	4 Weeks	8 Weeks	12 Weeks
Wrist flexor			
Liztox^®^ group	−1.14 ± 0.59	−1.02 ± 0.56	−0.96 ± 0.62
Botox^®^ group	−1.22 ± 0.59	−1.18 ± 0.60	−0.94 ± 0.64
Elbow flexor			
Liztox^®^ group	−0.98 ± 0.65	−0.89 ± 0.61	−0.81 ± 0.62
Botox^®^ group	−0.90 ± 0.65	−0.78 ± 0.62	−0.73 ± 0.59
Finger flexor			
Liztox^®^ group	−1.23 ± 0.73	−1.01 ± 0.64	−0.90 ± 0.70
Botox^®^ group	−1.26 ± 0.72	−1.19 ± 0.65	−0.97 ± 0.77

Change = weeks 4, 8, 12—baseline. There was no statistically significant difference between the two treatment groups at any time point (*p* > 0.05).

**Table 4:** Response rate of each injected muscle group at 4, 8, and 12 weeks post-injection by PPS.

Injection Muscle	Time after Injection	Response Rate (%)		*p*-Value
		Liztox^®^ Group	Botox^®^ Group	
Wrist flexor	4 weeks	69/84 (82.1)	70/82 (85.4)	0.5737
	8 weeks	63/82 (76.8)	64/79 (81.0)	0.5156
	12 weeks	57/81 (70.4)	54/80 (67.5)	0.6939
Elbow flexor	4 weeks	51/84 (60.7)	45/82 (54.9)	0.4465
	8 weeks	49/82 (59.8)	43/79 (54.4)	0.4948
	12 weeks	43/81 (53.1)	43/80 (53.8)	0.9327
Finger flexor	4 weeks	65/84 (77.4)	66/82 (80.5)	0.6237
	8 weeks	56/82 (68.3)	64/79 (81.0)	0.0640
	12 weeks	48/81 (59.3)	52/80 (65.0)	0.4528

*p*-value compares change from baseline between groups: Chi-square test. Evaluated by the MAS of the treatment site; a decrease of at least 1 point from baseline was defined as a responder.

**Table 5:** Change in the Disability Assessment Scale (DAS) scores for total and each item (PPS).

	Visits	Liztox^®^ Group			Botox^®^ Group			*p*-Value
		n	Mean ± SD	Median [Min, Max]	n	Mean ± SD	Median [Min, Max]	
Total								
	4 weeks	84	−1.19 ± 0.67	−1.00 [−2.00, 1.00]	82	−1.13 ± 0.72	−1.00 [−2.00, 0.00]	0.6045
	8 weeks	82	−1.12 ± 0.69	−1.00 [−2.00, 1.00]	79	−1.09 ± 0.72	−1.00 [−2.00, 1.00]	0.6881
	12 weeks	81	−1.14 ± 0.68	−1.00 [−3.00, 1.00]	80	−0.85 ± 0.60	−1.00 [−2.00, 1.00]	0.0047 *
Hand hygiene								
	4 weeks	18	−1.39 ± 0.50	−1.00 [−2.00, 1.00]	17	−1.24 ± 0.66	−1.00 [−2.00, 0.00]	0.5454
	8 weeks	18	−1.39 ± 0.61	−1.00 [−2.00, 0.00]	17	−1.12 ± 0.70	−1.00 [−2.00, 0.00]	0.2486
	12 weeks	18	−1.39 ± 0.61	−1.00 [−3.00, −1.00]	17	−0.76 ± 0.56	−1.00 [−2.00, 0.00]	0.0048 *
Dressing								
	4 weeks	2	−1.50 ± 0.71	−1.50 [−2.00, −1.00]	3	−0.67 ± 1.15	0.00 [−2.00, 0.00]	0.5428
	8 weeks	2	−1.50 ± 0.71	−1.50 [−2.00, −1.00]	3	−0.67 ± 1.15	0.00 [−2.00, 0.00]	0.5428
	12 weeks	2	−1.00 ± 1.41	−1.00 [−2.00, 0.00]	2	−1.00 ± 1.41	−1.00 [−2.00, 0.00]	1.0000
Limb position								
	4 weeks	61	−1.16 ± 0.69	−1.00 [−2.00, 1.00]	58	−1.16 ± 0.72	−1.00 [−2.00, 0.00]	0.9348
	8 weeks	59	−1.05 ± 0.71	−1.00 [−2.00, 1.00]	56	−1.13 ± 0.72	−1.00 [−2.00, 1.00]	0.6246
	12 weeks	58	−1.05 ± 0.69	−1.00 [−2.00, 1.00]	57	−0.88 ± 0.60	−1.00 [−2.00, 1.00]	0.1289
Pain								
	4 weeks	3	−0.33 ± 0.58	0.00 [−1.00, 0.00]	4	−0.75 ± 0.50	−1.00 [−1.00, 0.00]	0.4142
	8 weeks	3	−0.67 ± 0.58	−1.00 [−1.00, 0.00]	3	−0.67 ± 0.58	−1.00 [−1.00, 0.00]	1.0000
	12 weeks	3	−1.33 ± 0.58	−1.00 [−2.00, −1.00]	4	−0.75 ± 0.50	−1.00 [−1.00, 0.00]	0.2703

Change = week 4, 8, 12—baseline. *p*-value for comparisons between treatment groups: Wilcoxon rank sum test. * The level of significance was set at *p* < 0.05

**Table 6:** Global assessment of treatment from the PPS.

		Liztox^®^ Group		Botox^®^ Group		*p* Value
	Visits	*n*	Responder *n* (%)	*n*	Responder *n* (%)	
Investigator						
	4 weeks	84	76 (90.5)	82	71 (86.6)	0.4311
	8 weeks	82	69 (84.2)	79	66 (83.5)	0.9173
	12 weeks	81	69 (85.2)	80	63 (78.8)	0.2881
Participant/caregiver						
	4 weeks	84	58 (69.1)	82	53 (64.6)	0.5458
	8 weeks	82	57 (69.5)	79	46 (58.2)	0.1360
	12 weeks	81	50 (61.7)	80	46 (57.5)	0.5846

*p*-value for comparisons between treatment groups: Chi-square test. Defined as a responder if the Global Assessment Scale was 5 or higher.

**Table 7:** Changes from baseline in the Caregiver Burden Scale (PPS).

	Visits	Liztox^®^ Group		Botox^®^ Group				*p*-Value
		*n*	Mean ± SD	Median [Min, Max]	*n*	Mean ± SD	Median [Min, Max]	
Cleaning the palm								
	4 weeks	84	−0.43 ± 1.11	0.00 [−3.00, 2.00]	82	−0.39 ± 1.10	0.00 [−4.00, 2.00]	0.7674
	8 weeks	82	−0.43 ± 1.17	0.00 [−3.00, 3.00]	79	−0.65 ± 1.23	0.00 [−4.00, 2.00]	0.4478
	12 weeks	81	−0.40 ± 1.20	0.00 [−3.00, 3.00]	80	−0.59 ± 1.21	0.00 [−4.00, 2.00]	0.3341
Cutting fingernails								
	4 weeks	84	−0.57 ± 1.08	0.00 [−4.00, 2.00]	82	−0.50 ± 1.10	0.00 [−4.00, 3.00]	0.5299
	8 weeks	82	−0.52 ± 1.10	0.00 [−4.00, 2.00]	79	−0.65 ± 1.16	0.00 [−4.00, 1.00]	0.5993
	12 weeks	81	−0.44 ± 1.08	0.00 [−4.00, 3.00]	80	−0.63 ± 1.22	0.00 [−4.00, 1.00]	0.8490
Dressing								
	4 weeks	84	−0.43 ± 0.92	0.00 [−3.00, 2.00]	82	−0.48 ± 1.07	0.00 [−3.00, 3.00]	0.9716
	8 weeks	82	−0.38 ± 0.88	0.00 [−3.00, 2.00]	79	−0.52 ± 1.05	0.00 [−4.00, 2.00]	0.4358
	12 weeks	81	−0.41 ± 0.96	0.00 [−3.00, 2.00]	80	−0.59 ± 1.06	0.00 [−4.00, 1.00]	0.4908
Cleaning under the armpits								
	4 weeks	84	−0.44 ± 1.01	0.00 [−3.00, 2.00]	82	−0.60 ± 1.30	0.00 [−4.00, 3.00]	0.6434
	8 weeks	82	−0.51 ± 0.91	0.00 [−3.00, 2.00]	79	−0.54 ± 1.27	0.00 [−4.00, 3.00]	0.7002
	12 weeks	81	−0.42 ± 1.09	0.00 [−3.00, 2.00]	80	−0.59 ± 1.23	0.00 [−4.00, 2.00]	0.8390

*p*-value compares change from baseline between groups: Wilcoxon rank sum test. Change = week 4, 8, 12—baseline.

**Table 8:** Treatment-emergent adverse events (safety set).

System Organ Class/Preferred Term	Liztox^®^ Group (n = 98)	Botox^®^ Group (n = 100)	Total (n = 198)
Subjects with TEAEs, n (%) [event]	11 (11.22) [29]	13 (13.00) [17]	24 (12.12) [46]
Infections and infestations	3 (3.06) [5]	6 (6.00) [6]	9 (4.55) [11]
COVID-19	3 (3.06) [3]	4 (4.00) [4]	7 (3.54) [7]
Bacterial infection	0 (0.00) [0]	1 (1.00) [1]	1 (0.51) [1]
Bronchitis	1 (1.02) [1]	0 (0.00) [0]	1 (0.51) [1]
Otitis media	0 (0.00) [0]	1 (1.00) [1]	1 (0.51) [1]
Pneumonia	1 (1.02) [1]	0 (0.00) [0]	1 (0.51) [1]
Nervous system disorders	2 (2.04) [2]	4 (4.00) [4]	6 (3.03) [6]
Cerebral infarction	0 (0.00) [0]	1 (1.00) [1]	1 (0.51) [1]
Cerebrovascular accident	0 (0.00) [0]	1 (1.00) [1]	1 (0.51) [1]
Dizziness	1 (1.02) [1]	0 (0.00) [0]	1 (0.51) [1]
Headache	0 (0.00) [0]	1 (1.00) [1]	1 (0.51) [1]
Paresthesia	0 (0.00) [0]	1 (1.00) [1]	1 (0.51) [1]
Transient ischemic attack	1 (1.02) [1]	0 (0.00) [0]	1 (0.51) [1]
General disorders and administration site conditions	3 (3.06) [3]	1 (1.00) [1]	4 (2.02) [4]
Pyrexia	1 (1.02) [1]	1 (1.00) [1]	2 (1.01) [2]
Asthenia	1 (1.02) [1]	0 (0.00) [0]	1 (0.51) [1]
Peripheral oedema	1 (1.02) [1]	0 (0.00) [0]	1 (0.51) [1]
Injury, poisoning, and procedural complications	1 (1.02) [1]	2 (2.00) [2]	3 (1.52) [3]
Hand fracture	1 (1.02) [1]	1 (1.00) [1]	2 (1.01) [2]
Tooth fracture	0 (0.00) [0]	1 (1.00) [1]	1 (0.51) [1]
Investigations	2 (2.04) [8]	0 (0.00) [0]	2 (1.01) [8]
Alanine aminotransferase increased	2 (2.04) [4]	0 (0.00) [0]	2 (1.01) [4]
Aspartate aminotransferase increased	2 (2.04) [4]	0 (0.00) [0]	2 (1.01) [4]
Gastrointestinal disorders	2 (2.04) [5]	0 (0.00) [0]	2 (1.01) [5]
Constipation	1 (1.02) [1]	0 (0.00) [0]	1 (0.51) [1]
Diarrhea	1 (1.02) [1]	0 (0.00) [0]	1 (0.51) [1]
Dyspepsia	1 (1.02) [1]	0 (0.00) [0]	1 (0.51) [1]
Hemorrhoids	1 (1.02) [1]	0 (0.00) [0]	1 (0.51) [1]
Vomiting	1 (1.02) [1]	0 (0.00) [0]	1 (0.51) [1]
Metabolism and nutrition disorders	1 (1.02) [2]	1 (1.00) [1]	2 (1.01) [3]
Hypokalemia	1 (1.02) [2]	0 (0.00) [0]	1 (0.51) [2]
Diabetes mellitus	0 (0.00) [0]	1 (1.00) [1]	1 (0.51) [1]
Skin and subcutaneous tissue disorders	1 (1.02) [1]	1 (1.00) [1]	2 (1.01) [2]
Dermatitis	0 (0.00) [0]	1 (1.00) [1]	1 (0.51) [1]
Pruritus	1 (1.02) [1]	0 (0.00) [0]	1 (0.51) [1]
Blood and lymphatic system disorders	1 (1.02) [1]	0 (0.00) [0]	1 (0.51) [1]
Febrile neutropenia	1 (1.02) [1]	0 (0.00) [0]	1 (0.51) [1]
Musculoskeletal and connective tissue disorders	0 (0.00) [0]	1 (1.00) [1]	1 (0.51) [1]
Pain in extremity	0 (0.00) [0]	1 (1.00) [1]	1 (0.51) [1]
Neoplasms: benign, malignant, and unspecified (including cysts and polyps)	0 (0.00) [0]	1 (1.00) [1]	1 (0.51) [1]
Benign neoplasm of skin	0 (0.00) [0]	1 (1.00) [1]	1 (0.51) [1]
Respiratory, thoracic, and mediastinal disorders	1 (1.02) [1]	0 (0.00) [0]	1 (0.51) [1]
Oropharyngeal pain	1 (1.02) [1]	0 (0.00) [0]	1 (0.51) [1]

TEAEs = treatment-emergent adverse events, AEs = adverse events. Adverse events were coded using MedDRA Version 25.1. Adverse events are displayed as number of subjects, percentage of subjects, and number of events. Percentages are based on the number of subjects in the treatment group.

**Table 9:** Adverse drug reactions and serious adverse events (safety set analysis).

System Organ Class/Preferred Term	Liztox^®^ Group (n = 98)	Botox^®^ Group (n = 100)	Total (n = 198)
Subjects with ADRs, n (%) [event]	2 (2.04) [2]	1 (1.00) [1]	3 (1.52) [3]
General disorders and administration site conditions	1 (1.02) [1]	0 (0.00) [0]	1 (0.51) [1]
Asthenia	1 (1.02) [1]	0 (0.00) [0]	1 (0.51) [1]
Nervous system disorders	0 (0.00) [0]	1 (1.00) [1]	1 (0.51) [1]
Headache	0 (0.00) [0]	1 (1.00) [1]	1 (0.51) [1]
Skin and subcutaneous tissue disorders	1 (1.02) [1]	0 (0.00) [0]	1 (0.51) [1]
Pruritus	1 (1.02) [1]	0 (0.00) [0]	1 (0.51) [1]
Subjects with SAEs, n (%) [event]	2 (2.04) [3]	2 (2.00) [2]	4 (2.02) [5]
Nervous system disorders	1 (1.02) [1]	2 (2.00) [2]	3 (1.52) [3]
Cerebral infarction	0 (0.00) [0]	1 (1.00) [1]	1 (0.51) [1]
Cerebrovascular accident	0 (0.00) [0]	1 (1.00) [1]	1 (0.51) [1]
Transient ischemic attack	1 (1.02) [1]	0 (0.00) [0]	1 (0.51) [1]
Blood and lymphatic system disorders	1 (1.02) [1]	0 (0.00) [0]	1 (0.51) [1]
Febrile neutropenia	1 (1.02) [1]	0 (0.00) [0]	1 (0.51) [1]
Gastrointestinal disorders	1 (1.02) [1]	0 (0.00) [0]	1 (0.51) [1]
Vomiting	1 (1.02) [1]	0 (0.00) [0]	1 (0.51) [1]

ADRs = adverse drug reactions, SAEs = serious adverse events. Adverse events were coded using MedDRA Version 25.1. Adverse events are displayed as number of subjects, percentage of subjects, and number of events. Percentages are based on the number of subjects in the treatment group.

## Data Availability

The data used and/or analyzed in this study are available from the corresponding author upon reasonable request.

## Supplementary Materials

The following supporting information can be downloaded at: https://www.mdpi.com/article/10.3390/toxins15120697/s1, Figure S1: Injection dose and site of study medication.
