# Supplementary material for: A Randomized, Double-Blind, Active Control, Multicenter, Phase 3 Study to Evaluate the Efficacy and Safety of Liztox® versus Botox® in Post-Stroke Upper Limb Spasticity
Source: Toxins (Basel). 2023 Dec 12;15(12):697. doi: 10.3390/toxins15120697 (PMC10748261; doi:10.3390/toxins15120697)
Supplement: Supplementary file 1 [file toxins-15-00697-s001.zip › toxins-2732570-supplementary.pdf]

Supplementary Materials: A Randomized, Double-blind, Active control, Multicenter, Phase 3 Study to Evaluate the Efficacy and Safety of Liztox® versus Botox® in Post-Stroke Upper Limb Spasticity

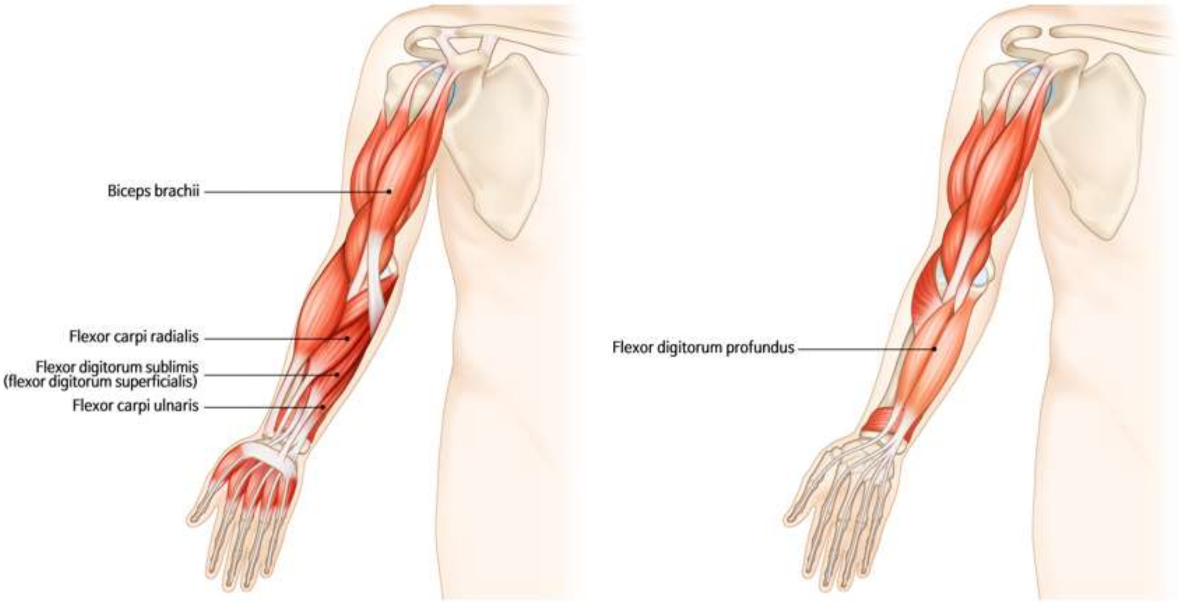

Muscle	Total dosage	Number of sites
Biceps brachii	100-200 U	Up to 4 sites
Flexor digitorum profundus	15-50 U	1-2 sites
Flexor digitorum sublimis	15-50 U	1-2 sites
Flexor carpi radialis	15-60 U	1-2 sites
Flexor carpi ulnaris	10-50 U	1-2 sites

Supplementary Figure S1. Injection dose and site of study medication.
